# Brain substrate metabolism and ß‐cell function in humans: A positron emission tomography study

**DOI:** 10.1002/edm2.136

**Published:** 2020-04-19

**Authors:** Eleni Rebelos, Andrea Mari, Marco Bucci, Miikka‐Juhani Honka, Jarna C. Hannukainen, Kirsi A. Virtanen, Jussi Hirvonen, Lauri Nummenmaa, Martin Heni, Patricia Iozzo, Ele Ferrannini, Pirjo Nuutila

**Affiliations:** ^1^ Turku PET Centre University of Turku Turku Finland; ^2^ Institute of Neuroscience National Research Council Padua Italy; ^3^ Clinical Nutrition Institute of Public Health and Clinical Nutrition University of Eastern Finland (UEF) Kuopio Finland; ^4^ Department of Radiology Turku University Hospital and University of Turku Turku Finland; ^5^ Department of Psychology University of Turku Turku Finland; ^6^ Department of Internal Medicine Division of Endocrinology Diabetology, Angiology, Nephrology and Clinical Chemistry Eberhard Karls University Tuebingen Tuebingen Germany; ^7^ Institute for Diabetes Research and Metabolic Diseases of the Helmholtz Center Munich at the University of Tuebingen Tuebingen Germany; ^8^ German Center for Diabetes Research (DZD e.V.) Neuherberg Germany; ^9^ Institute of Clinical Physiology National Research Council (CNR) Pisa Italy; ^10^ Department of Endocrinology Turku University Hospital Turku Finland

**Keywords:** brain glucose uptake, free fatty acids, glucose‐induced potentiation, insulin secretion, positron emission tomography

## Abstract

**Aims:**

Recent clinical studies have shown enhanced brain glucose uptake during clamp and brain fatty acid uptake in insulin‐resistant individuals. Preclinical studies suggest that the brain may be involved in the control of insulin secretion. The aim of this study was to investigate whether brain metabolism assessed as brain glucose and fatty acid uptake is associated with the parameters of β‐cell function in humans.

**Materials and methods:**

We analysed cross‐sectional data of 120 subjects across a wide range of BMI and insulin sensitivity. Brain glucose uptake (BGU) was measured during euglycaemic‐hyperinsulinaemic clamp (n = 67) and/or during fasting (n = 45) using [^18^F]‐fluorodeoxyglucose (FDG) positron emission tomography (PET). In another group of subjects (n = 34), brain fatty acid uptake was measured using [^18^F]‐fluoro‐6‐thia‐heptadecanoic acid (FTHA) PET during fasting. The parameters of β**‐**cell function were derived from OGTT modelling. Statistical analysis was performed with whole‐brain voxel‐based statistical parametric mapping.

**Results:**

In non‐diabetics, BGU during euglycaemic hyperinsulinaemic clamp correlated positively with basal insulin secretion rate (*r* = 0.51, *P* = .0008) and total insulin output (*r* = 0.51, *P* = .0008), whereas no correlation was found in type 2 diabetics. BGU during clamp correlated positively with potentiation in non‐diabetics (*r* = 0.33, *P* = .02) and negatively in type 2 diabetics (*r* = −0.61, *P* = .02). The associations in non‐diabetics were not explained with whole‐body insulin sensitivity or BMI. No correlations were found between baseline (fasting) BGU and basal insulin secretion rate, whereas baseline brain fatty acid uptake correlated directly with basal insulin secretion rate (*r* = 0.39, *P* = .02) and inversely with potentiation (*r* = −0.36, *P* = .04).

**Conclusions:**

Our study provides coherent, though correlative, evidence that, in humans, the brain may be involved in the control of insulin secretion independently of insulin sensitivity.

## Graphical abstract

Preclinical studies suggest that the brain may be involved in the control of insulin secretion, but data on humans are scanty. Our study provides coherent, though correlative, evidence that, in humans, the brain may be involved in the control of insulin secretion independently of insulin sensitivity.

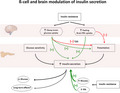

## 1 INTRODUCTION

The brain's role in the control of glucose metabolism is under extensive investigation. Preclinical research has provided evidence that both determinants of glucose tolerance, namely insulin secretion and insulin sensitivity, are in part modulated by the brain.^1^, ^2^, ^3^ Moreover, even though insulin sensitivity and insulin secretion are known to be tightly coupled,^4^, ^5^ the underlying coupling mechanisms have not been fully elucidated, with some researchers proposing an important role of the brain.^6^

With regard to insulin sensitivity, it has been shown that central insulin administration suppresses endogenous glucose production (EGP) in mice.^1^ Similarly, in humans, intranasal insulin delivery to the brain suppresses EGP in lean, but not in overweight, subjects.^7^ We have recently demonstrated that brain glucose uptake (BGU) during clamp associates positively with EGP in obese subjects, but not in lean controls,^8^ suggesting that adiposity modulates the brain's ability to control peripheral insulin sensitivity. It is of note that this association was found only during insulin stimulation, whereas in the fasting state, EGP and BGU were not found to be related. Furthermore, BGU has been found to be enhanced in obese subjects in comparison with lean subjects, only after insulin stimulation, whereas fasting BGU does not differ between obese and lean individuals.^9^ Similar findings of enhanced BGU during euglycaemic‐hyperinsulinaemic clamp, as compared to the fasting state, have been shown in both lean and obese minipigs.^10^ Thus far, the molecular mechanisms for these finding**s** have not been elucidated.

Preclinical studies in several species have provided evidence that the brain also modulates insulin secretion.^11^ Intracerebroventricular insulin administration stimulated insulin secretion in dogs.^2^ Studies in mice bearing a brain‐specific Glut2 deletion have provided further evidence suggesting a central control of insulin secretion. In this model, impairment of neuronal glucose sensing not only resulted in impaired cephalic and first‐phase insulin secretion, but also led to structural changes in the pancreas, such as reduced β‐cell mass.^12^ Recently, it has also been shown that cold exposure in rats leads to a ~ 50% decrease in insulin secretion and a doubling of insulin sensitivity. These effects were proposed to be mediated by the brain *via* the sympathetic nervous system, since administering α‐adrenergic receptor antagonist, phentolamine, fully reversed such effects.^13^ In a recent study in humans, using intranasal insulin administration, pancreatic insulin secretion was found to be reciprocally related to hypothalamic insulin sensitivity (defined as an increase in cerebral blood flow observed using functional magnetic resonance imaging).^14^

In humans, positron emission tomography (PET) is the gold standard technique for measuring regional metabolic rates in the brain and other tissues non‐invasively. ß‐cell function, on the other hand, can be reliably resolved using OGTT modelling.^15^ In this model, along with basal insulin secretion rate (ISR) and total insulin output, three major dynamic parameters are quantified: ß‐cell glucose sensitivity, rate sensitivity and potentiation of insulin secretion (reviewed in Ref. 15). Of particular interest is potentiation, that is the augmentation of insulin secretion in the presence of potentiating factors, since in addition to antecedent glycaemia and incretins, a neural factor has been suggested to partake in potentiation.

Thus, the aim of this study was to gain further insight into the brain‐pancreas axis in humans by examining the relationship between brain metabolism assessed using [^18^F]‐fluorodeoxyglucose (FDG) PET and ß‐cell function assessed through OGTT modelling. Since potentiation of insulin secretion is in part mediated by free fatty acids (FFA),^16^ we hypothesized that FFA might modulate any interactions between the brain and insulin secretion. To test this hypothesis, we additionally analysed data obtained in another group of subjects receiving PET with [^18^F]‐fluoro‐6‐thia‐heptadecanoic acid (FTHA), an analogue of palmitate.^17^

## 2 RESEARCH DESIGN AND METHODS

### 2.1 Study design and study population

We tested whether brain substrate uptake correlates with the parameters of β‐cell function using different data sets. All included studies were performed at Turku PET Centre during 2005‒2015. The anthropometric and metabolic characteristics of all study participants are listed in Table 1.

**Table 1:** Anthropometric characteristics and β‐cell function parameters of the study populations^a^

	Clamp FDG (a)		Fasting FTHA (b)		Fasting FDG (c)	
	Non‐T2D	T2D	Non‐T2D	T2D	Non‐T2D	T2D
M/F	9/43	4/11	0/26	0/8	10/29	0/6
Age (years)	45 ± 10	49 ± 7	44 ± 12	45 ± 8	44 ± 9	54 ± 5*
BMI (kg m^−2^)	27.4 [17.7]	34.0 [12.1]*	27.7 [17.8]	39.1 [8.0]*	32 [10.0]	41.5 [15.0]
HbA_1c_ (%), (mmol mol^−1^)	5.5[0.5], 37 [8]	5.9 [0.7], 41 [10]*	5.6 [0.6], 38 [6]	6.2 [1.0], 44 [11]*	5.8 [0.4], 40 [4]	6.8 [0.55], 51 [6]*
M‐value (μmol kg_FFM_^−1^ min^−1^)	39.4 [30.0]	19.3 [14.7]*	‐	‐	32.6 [32.6]	19.8 [23.3]
Serum insulin (pmol L^−1^)^b^	476 [221]	609 [161]*	40 [47]	119 [13]*	53 [44]	112 [63]*
Plasma glucose (mmol L^−1^)^b^	4.8 ± 0.4	4.9 ± 1.0	5.3 ± 0.5	7.0 ± 1.0*	5.7 ± 0.6	7.1 ± 2.3*
FFA (mmol L^−1^)^b^	0.05 [0.05]	0.1 [0.06]*	0.61 [0.40]	0.79 [0.44]	0.49 [0.31]	0.61 [0.26]
Basal ISR (pmol min^−1^ m^−2^)	86 [55]	135[38]*	81 [44]	152 [85]*	91 [66]	118 [72]
Total insulin output (nmol m^−2^)	40 [22]	48 [16]	44 [18]	44 [11]	‐	‐
ß‐GS (pmol^ ^min^−1^ m^−2^ mmol L^−1^)	119 [107]	48 [38]*	117 [101]	42 [40]*	‐	‐
Rate sensitivity (pmol m^−2^ mmol L^−1^)	506 [1363]	651 [1057]	922 [1091]	349 [410]*	‐	‐
Potentiation (ratio)	1.3 [0.7]	1.3 [1.1]	1.4 [1.6]	1.2 [0.8]	‐	‐
Brain substrate uptake (μmol 100 g^−1^ min^−1^)^#^	25.7 [10.9]	28.3 [8.0]*	0.99 [0.57]	1.1 [0.78]	20.5 [5.4]	17.5 [4.1]

^a^ Entries are mean ± SD or median [IQR]; ISR = insulin secretion rate; β‐GS = ß‐cell glucose sensitivity.

^b^ Serum insulin, serum FFA and plasma glucose levels that are presented are during the PET studies.

^#^ Glucose or FFA as appropriate for each dataset.

* *P* < .05, between T2D and non‐T2D.

In data set (a), we reanalysed all studies that had brain FDG‐PET scans carried out during a euglycaemic‐hyperinsulinaemic clamp and an OGTT (NCT00793143, NCT01344928) (n = 67).^8^, ^18^, ^19^, ^20^, ^21^ Data set (b) consisted of 34 women **(**20 morbidly obese and 14 age‐matched lean controls**)** who received FTHA‐PET during fasting and also had an OGTT (NCT01373892). Finally, in data set (c), we included subjects who were studied with brain FDG‐PET during fasting and had at least one fasting C‐peptide measurement (N = 45). Data sets (a) and (c) were partially overlapping since 20 subjects were studied both while fasting and during euglycaemic‐hyperinsulinaemic clamp using FDG‐PET. Figure S1 shows the PET protocols of the studies presented. In accordance with the ADA criteria,^22^ 15 subjects in data set (a), 8 in data set (b) and 6 in data set (c) were affected by T2D. Patients with T2D used only metformin (1‒3 g daily), and patients on insulin treatment were excluded. None of the study subjects had any clinical diagnoses of neurological diseases or psychiatric disorders. All subjects underwent a screening visit before inclusion in the study. During the visit, an OGTT was performed, with blood samples drawn at 0, 30, 60, 90 and 120 minutes for glucose, insulin and C‐peptide measurements**.** In data set (c), samples were drawn only at 0, 60 and 120 minutes, and thus, only the basal insulin secretion rate was calculated. Metformin was withheld 24‒72 hours before the metabolic studies. Prior to inclusion, each participant gave written consent. Each study protocol included in this study was approved by the Ethics Committee of the Hospital District of Southwest Finland and conducted in accordance with the Declaration of Helsinki.

### 2.2 PET study protocols

*Insulin clamp FDG studies:* The euglycaemic‐hyperinsulinaemic clamp was performed as previously described.^23^ In brief, the subjects were given a primed‐continuous infusion (40 mU m^−2^ min^−1^) of insulin (Actrapid; Novo Nordisk, Copenhagen, Denmark). During the clamp, a variable rate of a 20% glucose solution was infused in order to maintain euglycaemia (5 mmol/L). Plasma glucose levels were measured every 5‒10 min throughout the clamp. At 100 ± 10 min into the clamp, FDG (187 ± 9 MBq) was injected intravenously over a 15‐s period, and brain FDG radioactivity was measured either immediately thereafter or after 70‒80 min. For this reason, the time interval between the FDG injection and the brain scan was also used as a covariate in the statistical analysis. The PET study protocols are described in more detail in previous reports.^9^, ^24^ During the clamp, plasma glucose, plasma insulin and serum FFA were taken at baseline and every 5, 30 and 60 minutes, respectively.

*Fasting FDG studies*: During fasting, an intravenous bolus (188 ± 7 MBq) of FDG was given, following which the dynamic PET imaging of the brain started and continued for 40 minutes. Blood samples were drawn periodically during the entire scanning period to measure radioactivity levels.

*FTHA studies:* Following an overnight (10‒12 hours) fast, an intravenous bolus (185 ± 46 MBq) of FTHA was given, following which the dynamic PET imaging of the brain started and continued for 40 minutes. Blood samples were drawn during the entire scanning period to measure FFA as well as radioactivity levels.

### 2.3 Quantification of brain glucose and fatty acid uptake

Brain fractional uptake rate (FUR) of FDG was calculated by dividing the average level of brain radioactivity by the integral of the plasma radioactivity curve from the point of the FDG injection to the middle of the brain scan. More specifically, for those studies where brain radioactivity acquisition started immediately after FDG injection, the first 30 minutes was omitted and the selected time interval for calculating the average brain radioactivity was 30‒40 minutes. For the studies where brain radioactivity was measured at the end, the whole‐brain scan duration (15 minutes) was used. Brain glucose uptake (BGU, in μmol 100g^−1^ min^−1^) was then calculated at voxel level as follows: BGU = FUR.Cp/LC, where Cp is the average plasma glucose concentration from the injection until the end of the brain scan, and LC is the lumped constant for the brain (which was set at 0.65).^25^ Brain density was set at 1.04 g/mL.^26^ Brain FUR of FTHA was calculated by dividing brain radioactivity by the integral of the plasma unmetabolized radioactivity curve.^27^ Brain FFA uptake (BFAU, in μmol 100g^−1^ min^−1^) was then calculated at voxel level as follows: BFAU = FUR^.^ (serum FFA).

### 2.4 Analytical methods

Plasma glucose was measured in the laboratory of Turku PET Centre in duplicate using the glucose oxidase technique (Analox GM7 or GM9, Analox Instruments Ltd.). Glycosylated haemoglobin (HbA_1c_) was measured with ion‐exchange high‐performance liquid chromatography (Variant II Haemoglobin A_1c_, Bio‐Rad Laboratories). Serum insulin was determined by time‐resolved immunofluorometric assay (AutoDELFIA, PerkinElmer Life and Analytical Sciences). C‐peptide was measured with electrochemiluminescence analyser immunoassay (ECLIA) (Roche Diagnostics GmbH). Serum FFA were measured with a photometric enzymatic assay (FFA‐HR(2), Wako Chemicals GmbH) on Modular P800 automatic analyser (Roche Diagnostics). Detailed information regarding the analytical methods used can be found on the official website of Turku University Hospital (http://webohjekirja.mylabservices.fi/TYKS/).

### 2.5 Calculations

Insulin‐stimulated glucose disposal (M) was calculated as previously described^25^ and expressed per kg of fat‐free mass (μmol kg_FFM_^−1^ min^−1^), as this normalization has been shown to minimize differences in results caused by variations in sex, age and body weight.^28^

### 2.6 ß‐cell function

ß‐cell function was assessed from the OGTT using a model describing the relationship between insulin secretion rate (ISR, expressed in pmol min^−1^ m^−2^) and glucose concentration as the sum of two components.^29^, ^30^ The first component represents the dependence of ISR on glucose concentration through a dose‐response function relating the two variables. From the dose‐response, ß‐cell *glucose sensitivity* (the slope) was calculated. The dose‐response is modulated by a potentiation factor, accounting for various mechanisms (prolonged hyperglycaemia, non‐glucose substrates, gastrointestinal hormones, neural modulation). The potentiation factor averages 1 during the test and expresses relative potentiation or inhibition of ISR; its excursion is quantified by the ratio between the 2‐hour and the baseline value (*potentiation,* PFR: potentiation factor ratio). The second ISR component represents the dependence of ISR on the rate of change of glucose concentration and is determined by a single parameter (*rate sensitivity*), which is related to early insulin release. The model parameters were estimated from glucose and C‐peptide concentrations using C‐peptide deconvolution^31^ as previously described.^29^

### 2.7 Statistical analysis

Data are presented as mean ± SD (or median [IQR] for non‐normally distributed variables). Statistical analysis at the voxel and cluster levels was performed with statistical parametric mapping (SPM) (SPM12 toolbox for MATLAB). Linear regressions were performed in SPM to evaluate correlations between BGU (and BFAU) and single regressors (insulin secretion rate, potentiation), while controlling for confounding factors (in the SPM contrast, the controlling variables were set to a value of 0).

The statistical threshold in SPM analysis was set at a cluster level and corrected with false discovery rate (FDR) with *P* < .05. Glucose (and FFA) uptake values were extracted from nine regions of interest (ROI) (global, CER‐A: anterior cerebellum; CER‐P: posterior cerebellum; FRO: frontal lobe; LIMB: limbic lobe; MID: midbrain; OCC: occipital lobe; PAR: parietal lobe; TEMP: temporal lobe) with Marsbar plug‐in for MATLAB and correlated against the parameters of β‐cell function, using either Pearson (*r*) or Spearman (rho) correlation analysis, as appropriate.

Linear regression analysis was performed in order to evaluate possible confounding factors (time interval from FDG injection to the brain scan, scanner type, M‐value, BMI) in the found associations between BGU and the parameters of β‐cell function. Forward and backward search and Akaike's information criterion were used to find the optimal models for the study (the models are presented in detail in *Supplementary Data*).

Further statistical analyses were conducted using JMP version 13.0 (SAS Institute). A *P*‐value < .05 was considered statistically significant.

## 3 RESULTS

### 3.1 Brain glucose uptake during clamp and whole‐body glucose disposal

When compared to lean subjects, obese subjects had higher BGU during clamp in some but not all regions of interest examined. This finding is in accordance with previous findings (Table S1). In the pooled data from all subjects, BGU during clamp correlated negatively with the degree of insulin sensitivity (M‐value) (*r* = −0.40, *P* = .001) and positively with the average (steady‐state) FFA concentration (*r* = 0.42, *P* = .0007) (Figure S2A,B).

### 3.2 Brain glucose uptake and ß‐cell function

In the whole data set, there was a positive association between BGU during clamp and basal ISR (*r* = 0.44, *P* = .0002). Likewise, there was a positive association between BGU during clamp and total insulin output (*r* = 0.36, *P* = .003). Contrastingly, no significant association was found between fasting brain glucose uptake and basal ISR. With regard to the dynamic parameters of ß‐cell function, only potentiation (but not ß‐cell glucose sensitivity or rate sensitivity) was directly related to BGU (*r* = 0.24, *P* = .048).

**Figure 1:**
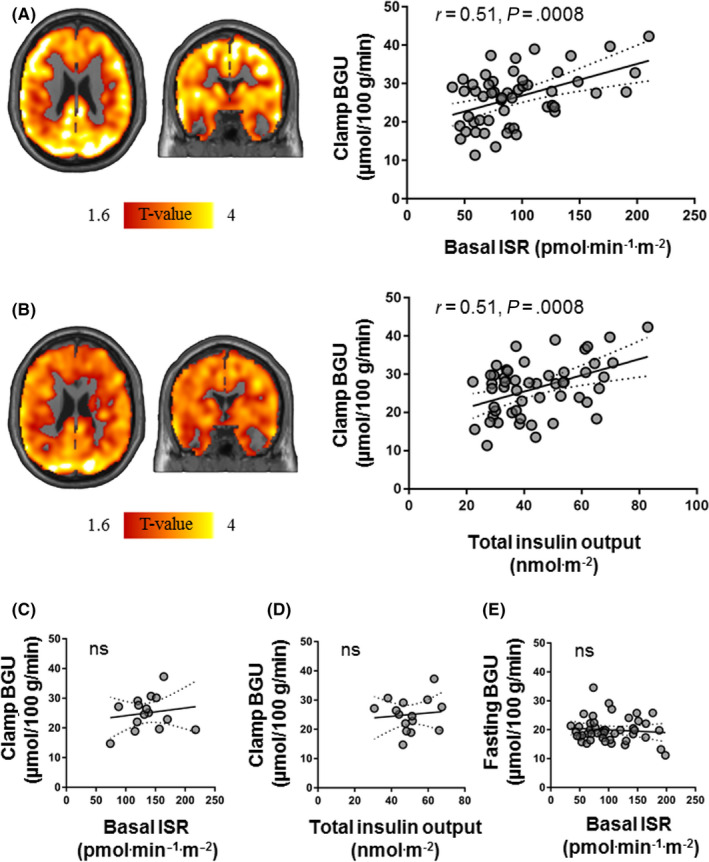
Brain clusters (as defined by FDR‐corrected SPM one‐sample t test) for the association between brain glucose uptake (BGU) during clamp and basal insulin secretion rate (ISR) (A) and total insulin output (B) in non‐diabetics. No correlation between BGU during clamp and basal insulin secretion rate (ISR) (C) or total insulin output in T2D (D). No correlation between fasting BGU and basal insulin secretion rate (E). For the corresponding scatterplots, the global ROI was extracted and used

**Figure 2:**
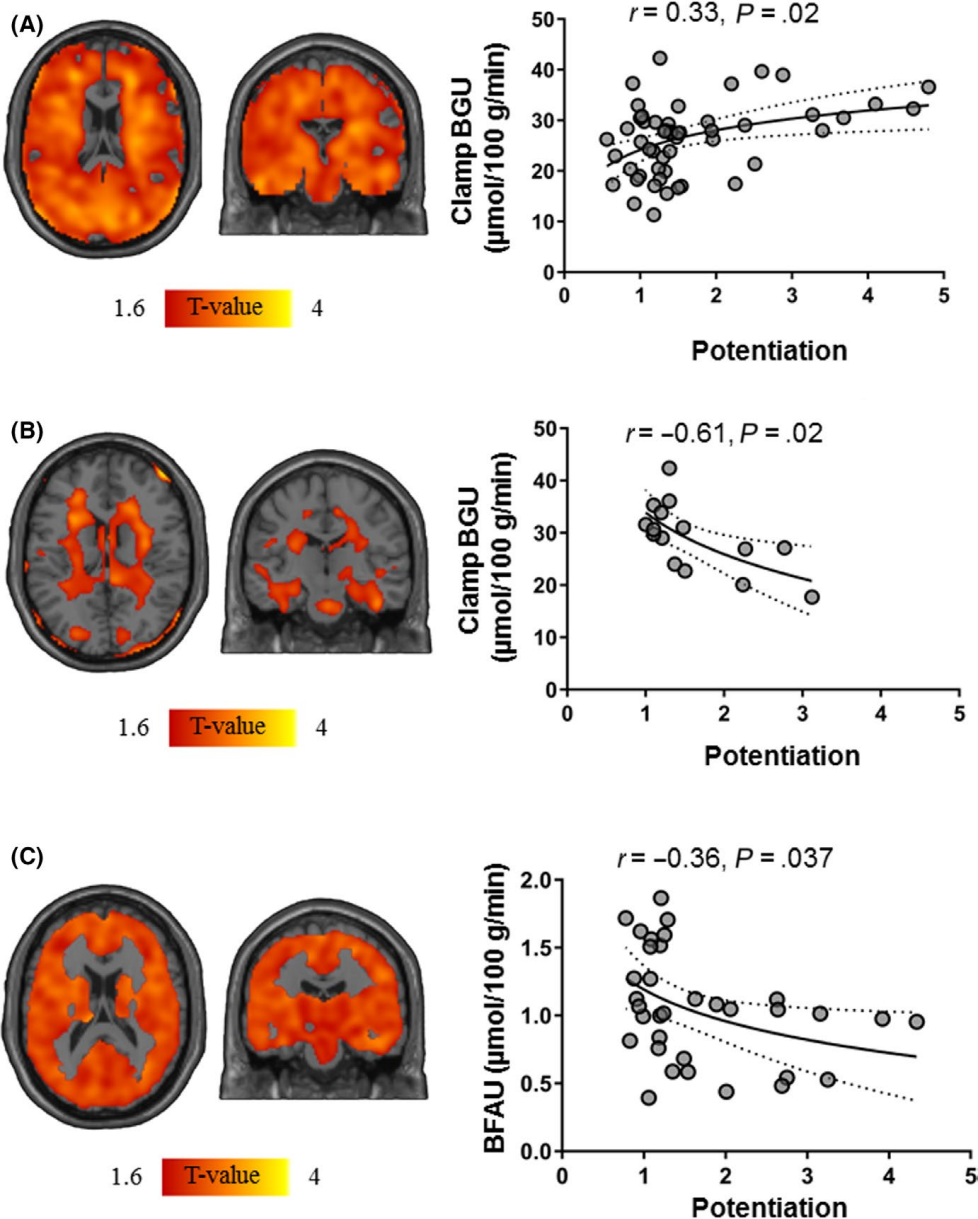
Brain clusters (as defined by FDR‐corrected SPM one‐sample t test) for the association between brain glucose uptake (BGU) during clamp and potentiation of insulin secretion in non‐diabetics (A) and T2D (B) and the corresponding scatterplots. Brain clusters (as defined by FDR‐corrected SPM one‐sample t test) for the association between brain fatty acid uptake (BFAU) and potentiation (C). Due to the non‐global effects, the occipital ROI was extracted and used in the scatterplots

The association between BGU and insulin secretion was driven by the non‐diabetic subjects, because when we divided the data set into two groups T2D (N = 15) and non‐T2D (N = 52), it remained significant only in the non‐T2D (*r* = 0.51, *P* = .0008 for basal ISR, and *r* = 0.51, *P* = .0008 for total insulin output) (Figure 1). However, potentiation behaved differently; and whereas BGU and potentiation were directly related in the 52 non‐T2D (*r* = 0.33, *P* = .02), in the T2D group there was a significant inverse correlation between BGU and PFR (*r*=−0.61, *P* = .02) (Figure 2).

In the non‐T2D group then only, we applied a stepwise selection approach in order to find the optimal models of prediction of BGU during clamp. In the models, we included possible confounders, such as the time interval from FDG injection to the brain scan, the fact that three different scanners were used, BMI, M‐value, steady‐state serum insulin levels and one parameter of ß‐cell function in use at the time. In all cases, the parameters of ß‐cell function (basal ISR, total insulin output and potentiation) were included in the optimal prediction models of BGU during clamp (Supplementary Data). In the same line, after partialling for insulin sensitivity (basal ISR, PFR) or BMI (for total insulin output), the associations between BGU and basal ISR (*r* = 0.32, *P* = .024), PFR (*r* = 0.44, *P* = .001) and total insulin output (*r* = 0.37, *P* = .008) remained significant.

### 3.3 Regional findings

In the 52 non‐T2D, the positive correlations between BGU and basal ISR as well as total insulin output were confirmed also in the various regions of interest examined (Table 2). The positive and negative correlations between BGU and PFR in non‐diabetics and in type 2 diabetics were also confirmed in most ROIs.

**Table 2:** Regional analysis of the associations between brain glucose uptake and basal insulin secretion rate (ISR), total insulin output (TIS) and potentiation

	BISR		TIS		Potentiation			
					non‐T2D		T2D	
	*P*‐value	*r*	*P*‐value	*r*	*P*‐value	Rho	*P*‐value	Rho
Cerebellum Anterior	.005	.39	.007	.37	.02	0.32	.02	−0.61
Cerebellum Posterior	.007	.37	.007	.37	.02	0.33	.04	−0.53
Occipital Lobe	.002	.43	.002	.43	.01	0.34	.02	−0.60
Parietal Lobe	.001	.45	.001	.45	.03	0.31	.1	−
Temporal Lobe	.003	.41	.005	.39	.03	0.30	.03	−0.56
Frontal Lobe	.002	.43	.002	.42	.03	0.30	.1	−
Limbic Lobe	.002	.42	.005	.39	.03	0.31	.03	−0.56
Midbrain	.002	.43	.004	.40	.04	0.30	.02	−0.61

Abbreviations: *r*, correlation coefficient; Rho, Spearman correlation coefficient.

### 3.4 Brain FFA uptake and ß‐cell function

As previously shown, brain FFA uptake (BFAU) was positively associated with BMI (*r* = 0.5, *P* = .003) and other measures of adiposity.^32^ BFAU also correlated with basal ISR (*r* = 0.39, *P* = .02) and, in an inverse manner, with potentiation (*r *= −0.36, *P* = .04) (Figure 2C). Similar trends of these associations were found also when dividing the population into non‐diabetics and type 2 diabetics (N = 8), even though the associations in the T2D group did not reach statistical significance probably because of low numerosity.

### 3.5 Regional findings

The correlations between BFAU and the aforementioned parameters of ß‐cell function were confirmed also in the various regions of interest examined (Table 3).

**Table 3:** Regional analysis of the associations between brain fatty acid uptake and basal insulin secretion rate (ISR) and potentiation

	Basal ISR		Potentiation	
	*P*‐value	*r*	*P*‐value	Rho
Cerebellum Anterior	.03	.38	.02	−0.41
Cerebellum Posterior	.04	.36	.04	−0.35
Occipital Lobe	.03	.38	.02	−0.41
Parietal Lobe	.02	.39	.01	−0.42
Temporal Lobe	.02	.39	.01	−0.43
Frontal Lobe	.02	.39	.01	−0.42
Limbic Lobe	.01	.42	.01	−0.42
Midbrain	.03	.38	.02	−0.39

Abbreviations: *r*, correlation coefficient; rho, Spearman correlation coefficient.

## 4 DISCUSSION

In this study, we examined the relationship between brain regional substrate utilization, as quantified by PET, and ß‐cell function, as resolved by mathematical modelling of OGTT data. In non‐diabetic subjects, we detected a positive association between basal and glucose‐stimulated insulin secretion and brain glucose uptake during clamp, whereas there was no such association observed in type 2 diabetics. On the contrary, potentiation of insulin release associated positively with brain glucose uptake during clamp in non‐T2D and negatively in T2D. No association between BGU and ß‐cell glucose sensitivity or rate sensitivity was detected. Finally, we found that brain FFA uptake correlated positively with basal insulin secretion rate and negatively with potentiation (similar trends were found in both T2D and non‐T2D). These results require specification.

Insulin secretion is physiologically coupled with insulin sensitivity. In response to insulin resistance, insulin secretion increases in order to maintain normal glucose homeostasis. Thus, the observed direct association between insulin secretion and brain glucose uptake may not at first glance be surprising, as both these parameters correlate with insulin sensitivity. However, the association between brain glucose uptake and insulin secretion remained statistically significant after accounting for the degree of insulin sensitivity or serum insulin levels. Even though potentiation of insulin release was not statistically different in T2D and non‐T2D in this data set, and it was unrelated to insulin sensitivity or any other parameter of ß‐cell function, there was a clearly different pattern of association with BGU during clamp in T2D and non‐T2D. Collectively, therefore, these results are compatible with the notion that ß‐cell function may be modulated by brain glucose uptake and that this effect is, at least in part, independent of insulin resistance. This suggestion is in line with growing evidence that points to an alternative course in the natural history of glucose intolerance, whereby insulin hypersecretion may occur independently of insulin resistance^33^, ^34^ as a result of influences deriving from the central nervous system.^35^ This control is lost or altered in case of T2D, thus a condition with established ß‐cell dysfunction.^36^

**Figure 3:**
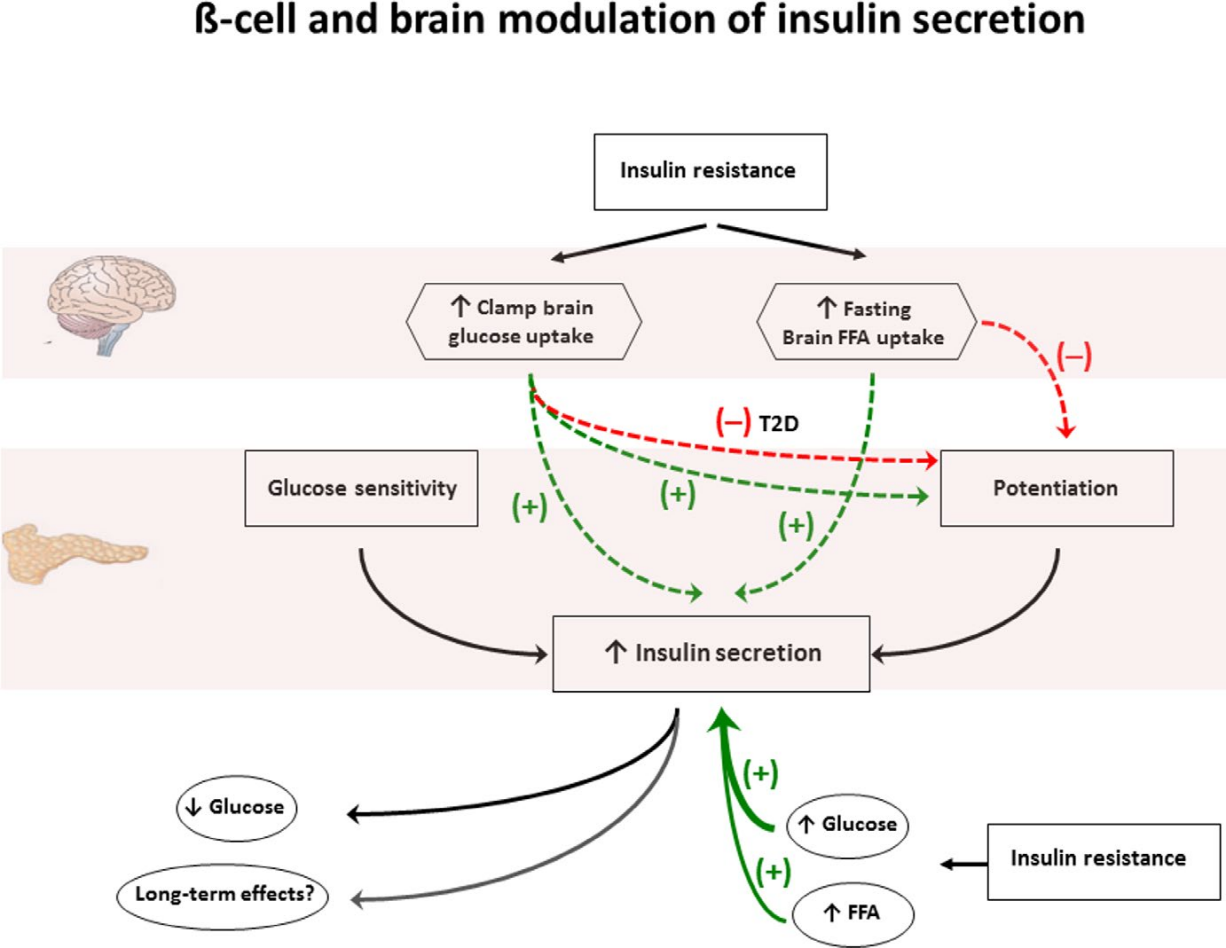
Schematic representation of the separate influence of substrate levels on insulin secretion, directly on the ß‐cell and indirectly via brain substrate uptake. Assuming that the brain ‘drives’ the observed correlations, increased BGU in conditions of insulin stimulation leads to enhanced insulin secretion in non‐diabetics and increased or decreased potentiation in non‐diabetics and T2D, respectively. Increased BFAU during fasting stimulates insulin secretion, but downregulates potentiation. The net effect of these phenomena would result in enhanced insulin secretion in non‐diabetics, which in the short term would restore normal plasma glucose levels. However, an activated brain‐pancreas axis might also have long‐term effects. In T2D, the brain control on insulin secretion seems either lost or reversed (see also text)

One possible mediator of the connection between BGU and insulin secretion could be FFA. In fact, we found circulating FFA to be positively linked to BGU, and FFA are known stimulators of pancreatic insulin secretion.^37^ FFA do enter the brain, and animal studies have suggested that hypothalamic sensing of circulating FFA is important in controlling nutrient intake and energy balance.^38^ Furthermore, we have previously shown that FFA uptake in the brain is enhanced in subjects with metabolic syndrome and is reduced following weight loss.^17^ This background prompted us to further investigate, whether FFA signalling in the brain could also contribute to the modulation of ß‐cell function. The current FTHA‐PET data show that brain FFA uptake under fasting conditions is directly associated with basal insulin secretion rate and negatively associated with potentiation. Thus, the uptake of glucose under insulinized conditions and the uptake of FFA under fasting conditions appear to be co‐stimulatory signals for insulin release in the brain. However, brain FFA uptake is a negative signal for potentiation of insulin release. This disassociation of substrate influence on insulin secretion *vs.* potentiation of insulin secretion is supported by recent data showing that potentiation of insulin release is impaired in insulin‐resistant individuals (who typically have elevated FFA levels)^39^ and that acute elevations of plasma FFA impair incretin‐induced potentiation of insulin release.^33^ The idea of a separate influence of substrate levels on insulin secretion—directly on the ß‐cell and indirectly *via* brain substrate uptake—is schematically summarized in Figure 3. Of course, brain modulation assumes that there exists a flexible neural outflow to the endocrine pancreas, as available evidence suggests.^40^

It is intriguing that associations between brain glucose uptake and insulin secretion along with potentiation were only found during insulin stimulation, whereas no association between BGU and insulin secretion was found in the fasting state. It is possible that the increased glucose availability to selected brain regions induced by insulin is read as a signal to upgrade global ß‐cell function in order to defend/restore normal glucose homeostasis. In the fasting state, on the other hand, the increased brain FFA uptake restrains ß‐cell function by downgrading potentiation. In both circumstances, brain substrate uptake associates positively with insulin resistance (or, equivalently, the BMI or FFA). This interpretation (outlined in Figure 3) is based on associations and is, therefore, speculative. However, there is supportive evidence in the literature. For instance, in rats 48‐h intracerebroventricular infusion of intralipid enhances glucose‐induced insulin secretion.^41^ Importantly, this brain‐pancreas axis could have beneficial short‐term effects restoring normal plasma glucose levels, but in the long term, it might ultimately contribute to the worsening of insulin resistance and/or ß‐cell overload (Figure 3).

Finally, we found no association between BGU and glucose sensitivity or rate sensitivity. However, glucose sensitivity and rate sensitivity quantify the dynamic insulin secretory response to an oral glucose stimulus, while basal ISR quantifies the tone of insulin secretion. As previously shown,^42^ basal insulin secretion and glucose‐stimulated insulin secretion have different characteristics and controls. It is thus not surprising that their association with brain glucose uptake does not involve all ß‐cell function parameters derived from OGTT modelling.

In recent years, the role of astrocytes has gained a lot of interest. Zimmer and colleagues demonstrated that the FDG uptake in the brain is driven by astrocytes.^43^ This finding, which is in accord with what was originally proposed as the astrocyte‐neuron lactate shuttle, whereby astrocytes convert glucose into lactate and supply lactate to neurons,^44^ may indicate that the increased BGU that we and others have constantly found in obesity,^9^ is at least in part due to astrogliosis. It has also been shown that astrocytes are the only cell type in the brain capable of oxidizing FFAs.^45^ Astrocytes express receptors for several hormones, such as leptin^46^ and insulin,^47^ and this observation has raised the possibility that astrocytes may participate in the regulation of energy homeostasis. Interestingly, astrocytes’ involvement in the control of both insulin and glucagon secretion has been shown after intracarotid injection of glucose and intracerebroventricular injection of 2‐deoxy‐D‐glucose, respectively.^48^, ^49^ Unfortunately, the poor spatial resolution of the PET (6‒8 mm) does not make it possible to distinguish the cell types involved in our findings. For the same reason, small brain areas, such as the hypothalamus, cannot be adequately addressed in humans with PET studies.^50^

There are further limitations in our study. The current analysis documents associations, but does not explain the mechanisms underlying the relationship between BGU (and BFAU) and the parameters of insulin secretion. Also, ß‐cell function was assessed after OGTT, whereas PET studies were performed on a separate day. Pooling data from smaller studies, which differed in their scanning designs, may also have added some noise in the results. To counter this, all data were reanalysed in identical manner and differences in the brain scanning times were carefully harmonized with appropriate modelling. Finally, due to the fact that, in Finland, women are more likely to undergo bariatric surgery than men, the FTHA data comprised of women only.

In conclusion, it has long been postulated that the brain is involved in the control of insulin secretion. Our study provides coherent, though correlative, evidence that in humans, the brain may be involved in the control of insulin secretion independently of insulin sensitivity. Further studies are warranted in the pursuit of unravelling underlying mechanisms as well as to evaluate the brain's contribution to ß‐cell failure in type 2 diabetes.

## CONFLICT OF INTEREST

No potential conflicts of interest relevant to this article were reported.

## AUTHORS’ CONTRIBUTION

E. R., MB, MJH, JCH, JH and LN analysed data and literature and drafted the manuscript. JCH conducted all clinical PET studies. MB and LN analysed the compartmental data analyses. AM performed OGTT modelling. PI, KAV and PN conceived the study designs. EF, AM, MH, PI and PN reviewed the manuscript. All authors approved the final version of the manuscript. PN is the guarantor of this work and, as such, had full access to all data in the study and takes responsibility for the integrity of the data as well as the accuracy of data analysis.

## PRIOR PRESENTATION

Parts of this study were presented as abstract at the Keystone Symposia (January 2018) and at the 54th EASD annual meeting at Berlin (October 2018).

## Supporting information

Figure S1

Figure S2

Data S1

## Data Availability

The data that support the findings of this study are available from the corresponding author upon reasonable request.
