# Supplementary figures and images for: Brain substrate metabolism and ß‐cell function in humans: A positron emission tomography study
Source: Endocrinol Diabetes Metab. 2020 Apr 19;3(3):e00136. doi: 10.1002/edm2.136 (PMC7375082; doi:10.1002/edm2.136)

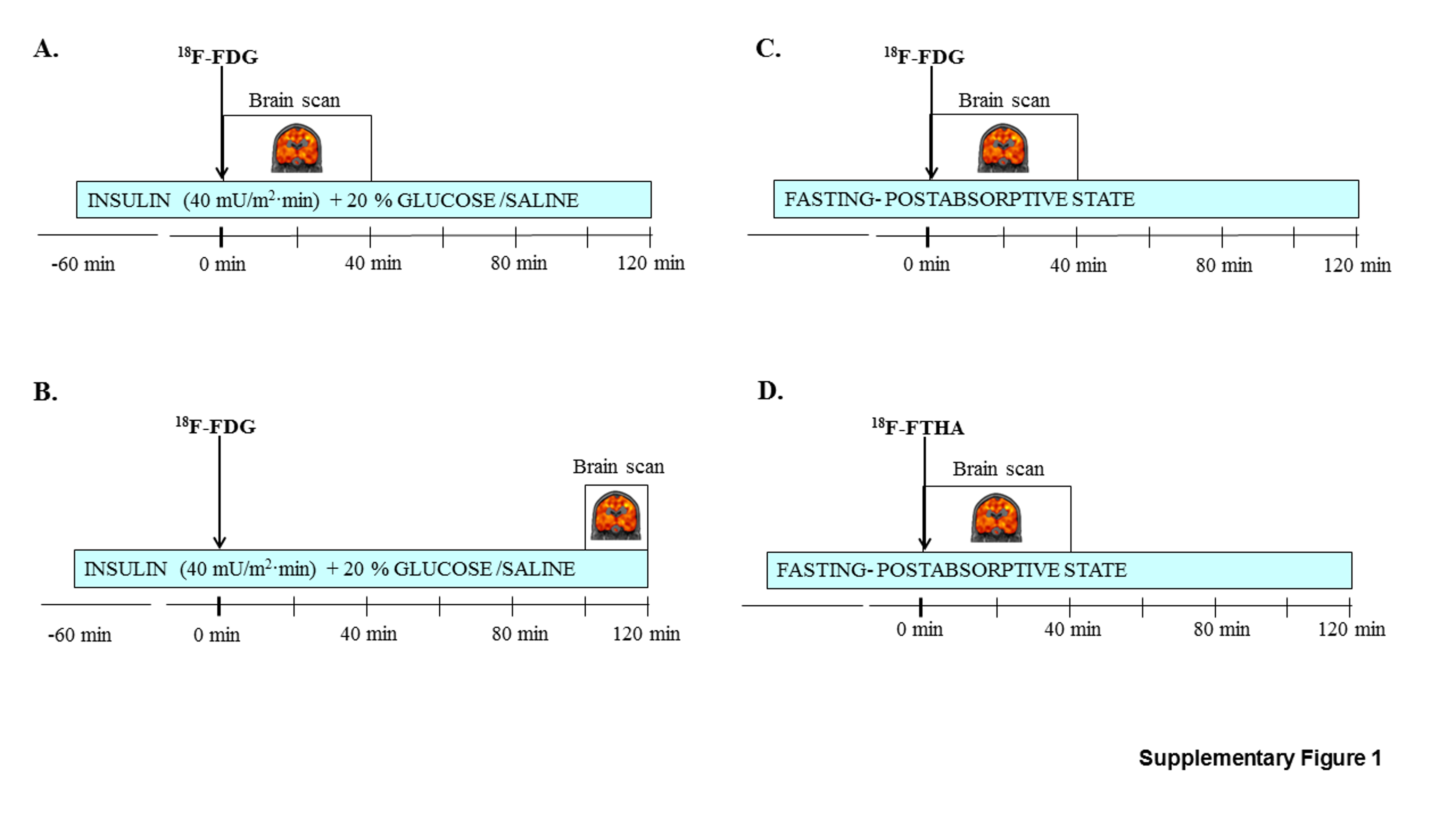

Supplement: Supplementary file 1 — Figure S1 [file EDM2-3-e00136-s001.tif]

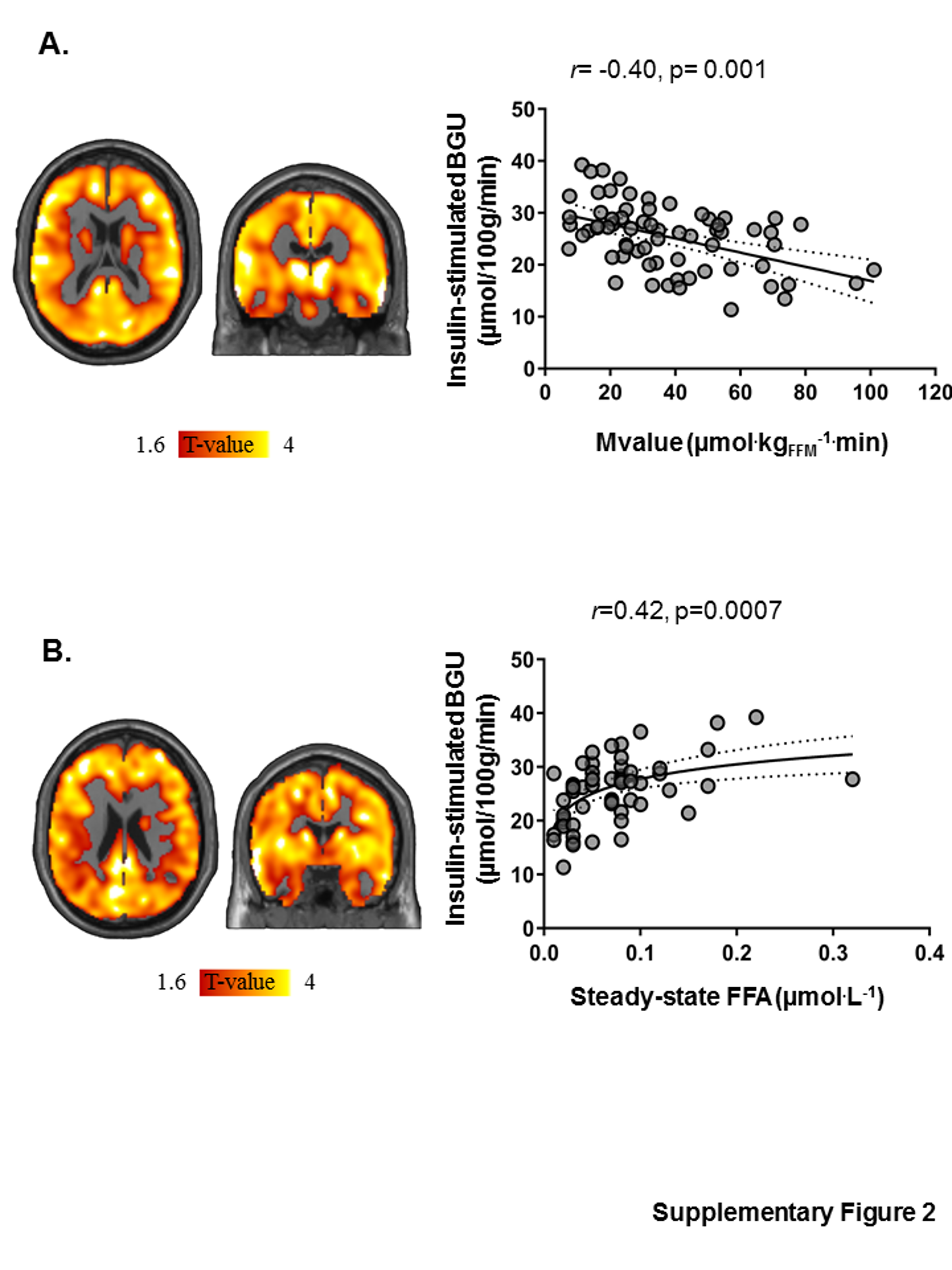

Supplement: Supplementary file 2 — Figure S2 [file EDM2-3-e00136-s002.tif]
