# Supplementary material for: Brain substrate metabolism and ß‐cell function in humans: A positron emission tomography study
Source: Endocrinol Diabetes Metab. 2020 Apr 19;3(3):e00136. doi: 10.1002/edm2.136 (PMC7375082; doi:10.1002/edm2.136)
Supplement: Supplementary file 3 — Data S1 [file EDM2-3-e00136-s003.docx]

**Supplementary data**

*Methods*

*Study population*

Apart from the information regarding T2D, already provided in the main text, some of the study participants were also affected by hypertension and dyslipidaemia. Medical treatments were ACE-inhibitors alone (10%), or in combination with thiazides (7%) for hypertension, and simvastatin for dyslipidaemia (9%).

*Results*

Models using Akaike’s information criterion predicting insulin-stimulated BGU by evaluating potentiation (**A**), basal insulin secretion rate (**B**) and total insulin output (**C**) and possible confounders.

**A**)

| **Lock** | **Entered** | **Parameter** | **Estimate** | **nDF** | **SS** | **"F Ratio"** | **"Prob>F"** |
| --- | --- | --- | --- | --- | --- | --- | --- |
| [x] | [x] | Intercept | 48.6140933 | 1 | 0 | 0.000 | 1 |
| [ ] | [ ] | BMI | 0 | 1 | 4.790315 | 0.321 | 0.57356 |
| [ ] | [x] | M value | -0.1319951 | 1 | 362.7308 | 24.705 | 0.00001 |
| [ ] | [x] | **Potentiation** | 1.41449681 | 1 | 83.99716 | 5.721 | **0.02091** |
| [ ] | [x] | SCANNER{SIEMENS HR&GE DSTE-GE ADVANCE} | 10.2078638 | 2 | 196.2934 | 6.685 | 0.00283 |
| [ ] | [x] | SCANNER{SIEMENS HR-GE DSTE} | -3.0707815 | 1 | 176.301 | 12.008 | 0.00116 |
| [ ] | [x] | Minutes from FDG | -0.3879297 | 1 | 65.91733 | 4.490 | 0.03953 |

| **Lock** | **Entered** | **Parameter** | **Estimate** | **nDF** | **SS** | **"F Ratio"** | **"Prob>F"** |
| --- | --- | --- | --- | --- | --- | --- | --- |
| [x] | [x] | Intercept | 48.6140933 | 1 | 0 | 0.000 | 1 |
| [ ] | [ ] | BMI | 0 | 1 | 4.790315 | 0.321 | 0.57356 |
| [ ] | [x] | M value | -0.1319951 | 1 | 362.7308 | 24.705 | 0.00001 |
| [ ] | [x] | **Potentiation** | 1.41449681 | 1 | 83.99716 | 5.721 | **0.02091** |
| [ ] | [x] | SCANNER{SIEMENS HR&GE DSTE-GE ADVANCE} | 10.2078638 | 2 | 196.2934 | 6.685 | 0.00283 |
| [ ] | [x] | SCANNER{SIEMENS HR-GE DSTE} | -3.0707815 | 1 | 176.301 | 12.008 | 0.00116 |
| [ ] | [x] | Minutes from FDG | -0.3879297 | 1 | 65.91733 | 4.490 | 0.03953 |
| [ ] | [ ] | steady-state serum insulin | 0 | 1 | 2.546776 | 0.170 | 0.68178 |

**B**)

| **Lock** | **Entered** | **Parameter** | **Estimate** | **nDF** | **SS** | **"F Ratio"** | **"Prob>F"** |
| --- | --- | --- | --- | --- | --- | --- | --- |
| [x] | [x] | Intercept | 33.5660369 | 1 | 0 | 0.000 | 1 |
| [ ] | [x] | BMI | -0.141709 | 1 | 40.99681 | 3.161 | 0.08189 |
| [ ] | [x] | M value | -0.0716249 | 1 | 62.37511 | 4.809 | 0.03329 |
| [ ] | [ ] | SCANNER{SIEMENS HR&GE DSTE-GE ADVANCE} | 0 | 1 | 5.37234 | 0.409 | 0.52564 |
| [ ] | [ ] | SCANNER{SIEMENS HR-GE DSTE} | 0 | 2 | 32.20328 | 1.255 | 0.29487 |
| [ ] | [x] | Minutes from FDG | -0.1293087 | 1 | 993.674 | 76.616 | 2e-11 |
| [ ] | [x] | **Basal insulin secretion rate** | 0.05239646 | 1 | 104.1299 | 8.029 | **0.00676** |

| **Lock** | **Entered** | **Parameter** | **Estimate** | **nDF** | **SS** | **"F Ratio"** | **"Prob>F"** |
| --- | --- | --- | --- | --- | --- | --- | --- |
| [x] | [x] | Intercept | 33.5660369 | 1 | 0 | 0.000 | 1 |
| [ ] | [x] | BMI | -0.141709 | 1 | 40.99681 | 3.161 | 0.08189 |
| [ ] | [x] | Mvalue | -0.0716249 | 1 | 62.37511 | 4.809 | 0.03329 |
| [ ] | [ ] | SCANNER{SIEMENS HR&GE DSTE-GE ADVANCE} | 0 | 1 | 5.37234 | 0.409 | 0.52564 |
| [ ] | [ ] | SCANNER{SIEMENS HR-GE DSTE} | 0 | 2 | 32.20328 | 1.255 | 0.29487 |
| [ ] | [x] | Minutes from FDG | -0.1293087 | 1 | 993.674 | 76.616 | 2e-11 |
| [ ] | [x] | **Basal insulin secretion rate** | 0.05239646 | 1 | 104.1299 | 8.029 | **0.00676** |
| [ ] | [ ] | steady-state serum insulin | 0 | 1 | 19.3632 | 1.509 | 0.22552 |

**C**)

| **Lock** | **Entered** | **Parameter** | **Estimate** | **nDF** | **SS** | **"F Ratio"** | **"Prob>F"** |
| --- | --- | --- | --- | --- | --- | --- | --- |
| [x] | [x] | Intercept | 34.8449148 | 1 | 0 | 0.000 | 1 |
| [ ] | [ ] | BMI | 0 | 1 | 5.941876 | 0.405 | 0.52757 |
| [ ] | [x] | SCANNER{SIEMENS HR&GE DSTE-GE ADVANCE} | 8.17433267 | 2 | 115.7009 | 3.996 | 0.02497 |
| [ ] | [x] | SCANNER{SIEMENS HR-GE DSTE} | -2.2937525 | 1 | 100.6791 | 6.954 | 0.0113 |
| [ ] | [x] | Minutes from FDG | -0.3493535 | 1 | 54.206 | 3.744 | 0.05903 |
| [ ] | [x] | **Total insulin output** | 0.19152558 | 1 | 396.9919 | 27.420 | **3.77e-6** |

| **Lock** | **Entered** | **Parameter** | **Estimate** | **nDF** | **SS** | **"F Ratio"** | **"Prob>F"** |
| --- | --- | --- | --- | --- | --- | --- | --- |
| [x] | [x] | Intercept | 34.8449148 | 1 | 0 | 0.000 | 1 |
| [ ] | [ ] | BMI | 0 | 1 | 5.941876 | 0.405 | 0.52757 |
| [ ] | [x] | SCANNER{SIEMENS HR&GE DSTE-GE ADVANCE} | 8.17433267 | 2 | 115.7009 | 3.996 | 0.02497 |
| [ ] | [x] | SCANNER{SIEMENS HR-GE DSTE} | -2.2937525 | 1 | 100.6791 | 6.954 | 0.0113 |
| [ ] | [x] | Minutes from FDG | -0.3493535 | 1 | 54.206 | 3.744 | 0.05903 |
| [ ] | [x] | **Total insulin output** | 0.19152558 | 1 | 396.9919 | 27.420 | **3.77e-6** |
| [ ] | [ ] | steady-state serum insulin | 0 | 1 | 14.45121 | 0.998 | 0.323 |

Technical Aspects

*PET data acquisition* The studies were performed after a 12-h fast using either the GE Advance PET camera (General Electric Medical Systems, Milwaukee, WI), or GE Discovery STE (General Electric Medical Systems, Milwaukee, WI, USA), or ECAT931/08 (Siemens Molecular Imaging, Inc., Knoxville, TN, USA). The scanners were cross-calibrated against the same VDC-404 Dose calibrator (COMECER Netherlands, Joure, the Netherlands) to ensure consistency of results. The productions FTHA and FDG have been described previously [1, 2]. All data were corrected for dead time, decay, and measured photon attenuation, and reconstructed using a Hann filter with a cut-off frequency of 0.5 and a median root prior reconstruction method [3]. Arterialized blood samples were drawn during the scan and analysed for radioactivity concentration in plasma using an automatic γ counter (Wizard 1480, Wallac, Turku, Finland).

*Image preprocessing:* All studies were pre-processed similarly via the internally developed pipeline named MAGIA (<http://emotion.utu.fi/softwaredata/>, https://github.com/tkkarjal/magia). Dynamic PET images were motion corrected and mean images were calculated. Mean PET images were spatially normalized to FDG template in Montreal Neurological Institute (MNI) space (MNI International Consortium for Brain Mapping) using SPM12 ([www.fil.ion.ucl.ac.uk/spm/](http://www.fil.ion.ucl.ac.uk/spm/)) running on Matlab for Linux (version 9.1.0; Math Works, Natick, MA). Normalization parameters were subsequently applied to corresponding dynamic images and Ki (influx constant rate) parametric images were calculated. Parametric images were smoothed at 8 mm full-width at half-maximum.

A global and eight brain regions of interest (CER-A, anterior cerebellum; CER-P, posterior cerebellum; FRO, frontal lobe; LIMB, limbic lobe; MID, midbrain; OCC, occipital lobe; PAR, parietal lobe; TEMP, temporal lobe) were selected *via* wfu pickatlas (www.fmri.wfubmc.edu/cms/software) and extracted from the BGU parametric images via Marsbar (<http://marsbar.sourceforge.net)> in order to perform the linear regressions with the various predictors.

References

1. Hovik R, Osmundsen H, Berge R, et al (1990) Effects of thia-substituted fatty acids on mitochondrial and peroxisomal beta-oxidation. Studies in vivo and in vitro. Biochem J 270(1):167-173. https://doi.org/[10.1042/bj2700167](https://doi.org/10.1042/bj2700167)
2. Hamacher K, Coenen HH, Stocklin G (1986) Efficient stereospecific synthesis of no-carrier-added 2-[18F]-fluoro-2-deoxy-D-glucose using aminopolyether supported nucleophilic substitution. J Nucl Med 27(2):235-238.
3. Alenius S, Ruotsalainen U (1997) Bayesian image reconstruction for emission tomography based on median root prior. Eur J Nucl Med 24(3):258-265. https://doi.org/10.1007/BF01728761

**Supplementary Table 1:** Differences in insulin-stimulated BGU between obese and lean subjects across the regions of interest examined

|  | **Obese** | **Lean** | ***p*** |
| --- | --- | --- | --- |
| Cerebellum Anterior GU (µmol/100g/min) | 22.1 ± 4.0 | 19.2 ± 3.7 | 0.01 |
| Cerebellum Posterior GU (µmol/100g/min) | 20.6 ± 4.0 | 18.2 ± 3.6 | 0.04 |
| Occipital Lobe GU (µmol/100g/min) | 23.9 ± 4.1 | 21.5 ± 4.4 | 0.04 |
| Parietal Lobe GU (µmol/100g/min) | 23.3 ± 4.1 | 22.5 ± 4.9 | ns |
| Temporal Lobe GU (µmol/100g/min) | 21.4 ± 3.5 | 20.0 ± 3.9 | ns |
| Frontal Lobe GU (µmol/100g/min) | 20.9 ± 3.6 | 20.6 ± 4.3 | ns |
| Limbic Lobe GU (µmol/100g/min) | 22.0 ± 3.9 | 20.4 ± 3.9 | ns |
| Midbrain GU (µmol/100g/min) | 20.5 ± 3.4 | 18.1 ± 3.5 | 0.02 |

* Entries are mean±SD. GU: glucose uptake

**Supplementary Figure 1**: Schematic diagrams of the study designs; A, B: FDG-PET studies during euglycaemic insulin clamp (dataset a); C: FDG-PET during fasting (dataset b); and D: FTHA-PET during fasting (dataset c).

**Supplementary Figure 2**.– Brain clusters (as defined by FDR-corrected SPM one-sample t-test) for the association between insulin-stimulated brain glucose uptake (BGU) and M value (A) and steady-state FFA (B). For the corresponding scatterplots, the global ROI was extracted and used.
